# Estrogen Inhibits Colon Polyp Formation by Reducing Angiogenesis in a Carcinogen-Induced Rat Model

**DOI:** 10.1155/2013/453898

**Published:** 2013-11-14

**Authors:** Jia Yang, Li-juan Xiong, Fei Xu, Xiang Zhao, Bo Liu, Kai-Lin Cai, Guo-bin Wang

**Affiliations:** ^1^Department of Gastrointestinal Surgery, the Union Hospital of Tongji Medical college of Huazhong University of Science & Technology, Wuhan 430022, China; ^2^Department of Infectious Diseases, the Union Hospital of Tongji Medical college of Huazhong University of Science & Technology, Wuhan 430022, China

## Abstract

*Objective.* To study the effects of estrogen on colon polyp formation, proliferation, and angiogenesis on a rat model of colon cancer induced by dimethylhydrazine (DMH). *Methods.* Thirty-six female ovariectomized (OVX) rats were randomly divided into 3 groups: (I) control group (administrated with vehicles weekly), (II) DMH group (administrated with DMH weekly), and (III) DMH + E_2_ group (administrated with DMH and 17*β*-estradiol weekly). The incidence, volumes, and multiplicity of colon polyps in each group were evaluated. The microvessel density (MVD), the expressions of Proliferating Cell Nuclear Antigen (PCNA), and the expressions of HIF-1**α** and VEGF in polyps were detected in each group. *Results. *Estrogen reduced the multiplicity, volumes, and the PCNA expressions of DMH-induced colon polyps. The MVD in DMH + E_2_ group was significantly lower than that in DMH group. Estrogen treatment decreased the HIF-1**α** and VEGF expressions at both mRNA and protein level. *Conclusion.* Estrogen replacement was protective for ovariectomized rats from DMH-induced carcinogenesis, and one of the mechanisms for this was due to estrogen's inhibitive effects on blood vessel formation by downregulating VEGF and HIF-1**α** expressions.

## 1. Introduction

Plenty of epidemiologic evidence demonstrated that estrogen might influence the incidence of colon cancer in women [1–3]. Colon cancer risk increased after menopause and decreased after hormone replacement treatment (HRT) [4]. Many hypotheses had been proposed and studied. Estrogen receptors were found in colon epithelium and the estrogen receptor beta was the dominant subtype [5]. On cell models, many studies had found that estrogen could affect the growth of cells originated from colon mucosa [6, 7]. On an animal model of rats induced by DMH, we have found that ovariectomy could promote colon tumor formation [8]. Since the angiogenesis was vital for tumorigenesis and the estrogen was a well-known vasoactive hormone, it was worth investigating whether estrogen could influence angiogenesis in the course of colon carcinogenesis.

There were several types of vasculation during carcinogenesis, including angiogenesis, vasculogenesi, and vasculogenic mimicry. In early stage of cancer, the main type of vessel formation was angiogenesis [9], triggered by proangiogenic factors [10]. Among the pro-angiogenic factors, VEGF was the essential factor in angiogenesis [11, 12]. In the present study, we studied the effects of estrogen on the microvessel density (MVD) and the expression of VEGF and its main upstream regulator HIF-1*α*.

## 2. Material and Methods

### 2.1. Chemicals and Reagents

DMH and 17*β*-estradiol were purchased from Sigma (St. Louis, MO).

### 2.2. Animals

Female Sprague-Dawley rats were purchased from the Animal Center of Tongji Medical College. Protocols for animal experimentation and maintenance were approved by the Animal Ethics Committee at our university and carried out in accordance with the institutional guidelines.

Thirty-six female rats (10 weeks of age) were housed in plastic cages (4 rats per cage) under standard laboratory conditions (21 ± 1°C temperature, 50 ± 10% humidity, and 12 h of light time from 6 am to 6 pm) with normal food and tap water provided *ad libitum*. All rats were ovariectomized (OVX) at the age of 11 weeks. At the age of 12 weeks, these rats were randomly divided into 3 groups according to the following treatment: control group (*n* = 12), DMH group (*n* = 12), and DMH + E_2_ group (*n* = 12). In the control group, rats were subcutaneously and intraperitoneally administrated with vehicles once a week. In the DMH group, rats received intraperitoneal injections of DMH (20 mg/kg body weight) [13, 14] once a week. In the DMH + E_2_ group, rats received subcutaneous injections of 17*β*-estradiol (40 *μ*g/kg body weight and dissolved in camellia oil) [15] once a week, together with the weekly intraperitoneal injections of DMH (20 mg/kg body weight). The weekly drugs injection lasted for 16 weeks. Six weeks after the last injection, all the animals were sacrificed by an overdose injection of chloral hydrate (600 mg/kg intraperitoneal injection).

### 2.3. Harvesting of Specimen

After sacrifice, the entire colorectums were collected and opened longitudinally and washed with PBS. Polyps were identified through visual macroscopic examination and later verified with histopathological examination. The location and number of all the polyps were recorded. The length (*L*), width (*W*), and height (*H*) of each polyp were measured, and the volume of each polyp was calculated using the formula *V* = *L* × *W* × *H* × *π*/6. The polyps with the volume ranging from 50 mm^3^ to 70 mm^3^ were cut into halves. One portion of the polyp was stored at −80°C for RT-PCR and Western blot, and the other half was fixed in 4% paraformaldehyde and embedded in paraffin block. The polyps with a volume of <50 mm^3^ or >70 mm^3^ were all fixed in 4% paraformaldehyde and embedded in paraffin block.

### 2.4. Histopathological Evaluation

Polyps were fixed in 4% paraformaldehyde and embedded in paraffin. Then the polyp samples were cut into 4 *μ*m sections using a microtome.

Sections were stained with hematoxylin and eosin (H & E) and examined histologically in a blinded manner.

### 2.5. Immunohistochemistry and MVD Assessment

Sections (4 *μ*m) were cut from paraffin-embedded polyp samples and mounted on poly-L-lysine-coated slides. Immunohistochemical staining was performed using anti-CD34 antibody (BOSTER, China) and anti-PCNA antibody (Cell Signaling technology, USA) with the avidin-biotin-peroxidase complex (ABC) method. The proliferation rate was assessed by the PCNA index, defined as the percentage of PCNA-positive cells. PCNA index was determined by counting PCNA-positive cells in a total of at least 1000 cells in different randomly selected areas at ×400 magnification. MVD was assessed by the method of Weidner [16]. The CD34-stained sections were initially scanned at low power (×40 and ×100) and the areas of specimens with the highest neovascularization stained by CD34 were selected as hot spots. Subsequently, microvessel counting was carried out in four fields of the hot spots at ×400 magnification. Any brown-stained endothelial cells or cell cluster clearly separated from adjacent microvessels, tumour cells, and other connective tissue elements were considered as a single countable vessel. Red blood cell or vessel lumen was not necessary to define a microvessel.

### 2.6. Real-Time Quantitative RT-PCR

Tissue samples from the polyps ranging in volume from 50 mm^3^ to 70 mm^3^ and the normal colonic mucosa samples in the control group were used. Total tissue RNA was extracted with Trizol reagent (Invitrogen) following the manufacture's instruction. The cDNAs from total RNA were synthesized using PrimeScript RT reagent Kit (Takara, Japan). The mRNA expression was evaluated by real-time PCR with an ABI StepOne Plus (Applied Biosystems, Singapore). GAPDH was applied as the internal control. The concentrations of the reagents were adjusted to reach a final volume of 20 *μ*L, containing 2 *μ*L cDNA product, 10 *μ*L SYBR Premix Ex Taq II (Takara, Japan), and 0.8 *μ*L of forward and reverse primers. The reaction was carried out by 45 amplification cycles of 95°C for 5 s and 60°C for 30 s. PCR primers were designed by Primer 5.0 and Blast search to check specificity. Primer sequences used are listed in Table 1. The results were calculated by 2^−ΔΔct^ method.

### 2.7. Western Blot

Tissue samples from the polyps ranging in volume from 50 mm^3^ to 70 mm^3^ and the normal colonic mucosa samples in the control group were used. Protein was extracted with protein extraction kit (Beyotime, China), separated on 10% SD-SPAGE, and transferred to polyvinylidene difluoride (PVDF) membranes (Millipore, USA). Membranes were blocked in 5% nonfat milk diluted in TBST for 1 hour at room temperature and then were incubated overnight at 4°C with the following primary antibodies: anti-HIF-1*α* (Abcam, Cambridge, UK) and anti-VEGF-A (Abcam, Cambridge, UK). After rinsing with TBST for three times, the membrane was incubated with horseradish peroxidase-conjugated secondary antibodies for 1 hour at room temperature. The outcome was visualized by the ECL Plus Western blotting detection system according to the manufacturer's instructions.

### 2.8. Statistical Analysis

Results are expressed as mean ± SEM. Data were evaluated by ANOVA in which multiple comparisons were performed using the least-significant difference method, while those data in heterogeneity of variance were analyzed by Kruskal-Wallis test. The volumes of polyps in the two experimental groups were evaluated by student's *t* test. Colon polyp incidence was expressed as percentages, and results were statistically analyzed using the chi-square test. The differences were considered statistically significant at *P* < 0.05. All analysis was tested with SPSS version 18.0.

## 3. Results

### 3.1. Estrogen Reduced the Multiplicity and Volumes of DMH-Induced Polyps and the PCNA Index

**Figure 1:**
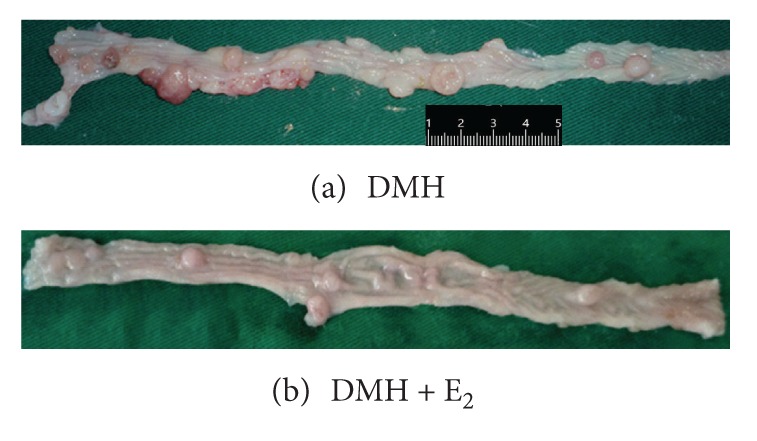
Polyps induced by DMH in experimental groups. (a) The polyps in DMH group. (b) The polyps in DMH + E_2_ group.

Eleven out of 12 (91.7%) rats in DMH group and 8 out of 12 (66.7%) rats in DMH + E_2_ group developed colon polyps (Figure 1), while none of the rats in control group developed colon polyps. The incidence of colon polyps in DMH group was higher than that in DMH + E_2_ group (91.7% versus 66.7%), though this difference was not statistically significant (*P* > 0.05) (Table 2). The polyp multiplicity (mean number of polyps per rat) in DMH group was significantly higher than that in DMH + E_2_ group (6.8 ± 3.1 versus 3.0 ± 1.1, *P* < 0.05) (Table 2). The polyps were mainly distributed in the distal colon compared to the proximal counterpart. The outcome was coincident with the animal model as Tanaka described [17]. At the same time, the average volume of polyps in DMH group was significantly bigger than that in DMH + E_2_ group (102.97 ± 77.67 versus 45.85 ± 43.40, *P* < 0.05) (Table 2).

**Figure 2:**
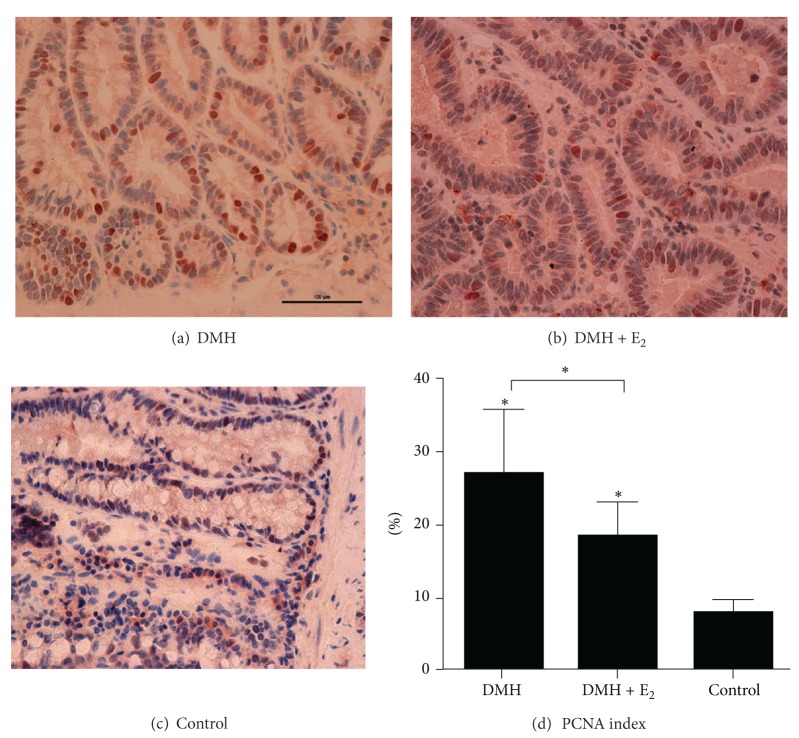
PCNA expression in each group. (a) PCNA staining in polyp from the DMH group. (b) PCNA staining in polyp from the DMH + E_2_ group. (c) PCNA staining in normal colonic mucosa from the control group. (d) Comparison of PCNA index in different groups (*P < 0.05).

The proliferation rates in different groups were assessed by the PCNA index. The PCNA index of the polyps from DMH group ranged from 19.6% to 31.2%, with an average of 27.1% ± 5.2%. When DMH was administrated together with estradiol in DMH + E_2_ group, the PCNA index decreased significantly to an average of 18.5% ± 2.9% (27.1% ± 5.2% versus 18.5% ± 2.9%, *P* < 0.05). The PCNA index in control group was significantly lower than that in the other two groups (Figure 2).

### 3.2. Estrogen Reduced the MVD in Polyps Induced by DMH

**Figure 3:**
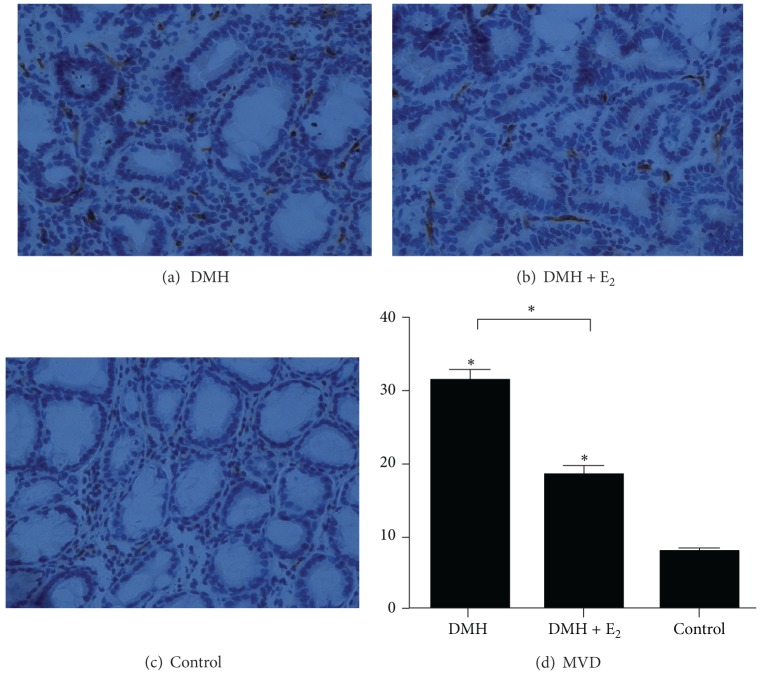
The MVD in each group. (a) Representative CD34 staining in polyp from the DMH group (×400 magnification). (b) Representative CD34 staining in polyp from the DMH + E_2_ group. (c) Representative CD34 staining in normal colonic mucosa from the control group. (d) Comparison of MVD in different groups (*P < 0.05).

The MVD in control group was significantly lower than that in the other two groups (Figure 3). The MVD elevated to an average of 32.13 ± 3.98 per field in DMH group. When DMH was administrated together with estradiol, the MVD decreased to 19.0 ± 4.24 per field. The MVD in DMH + E_2_ group was significantly lower than that in DMH group (*P* < 0.05) (Figure 3).

### 3.3. Estrogen Treatment Decreased the HIF-1*α* and VEGF Expressions at Both mRNA and Protein Level

**Figure 4:**
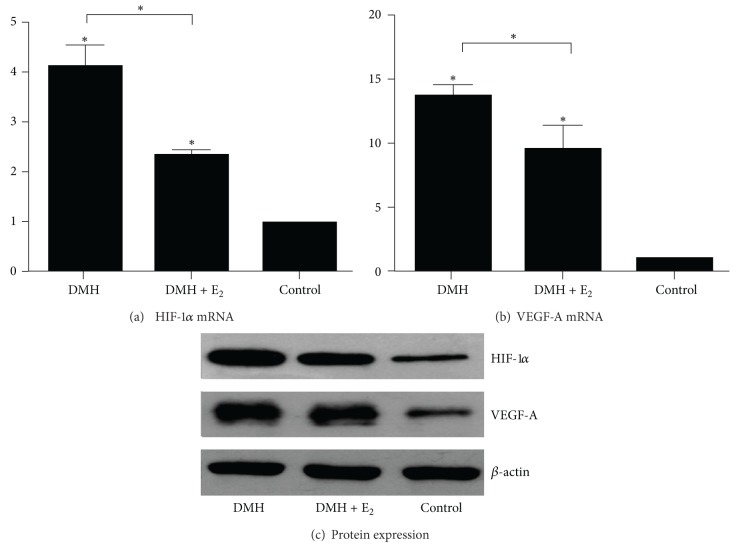
Expressions of HIF-1*α* and VEGF at mRNA and protein level. (a) Comparison of HIF-1*α* mRNA level in different groups (*P < 0.05). (b) Comparison of VEGF-A mRNA level in different groups (*P < 0.05). (c) Representative immunoblot analysis of HIF-1*α* and VEGF-A protein expressions in each group.

The mRNA expression of HIF-1*α* in the DMH group or DMH + E_2_ group was significantly upregulated compared to that of the control group (Figure 4(a)). And the mRNA transcripts in DMH group were almost 2-fold higher than that in DMH + E_2_ group (Figure 4(a); *P* < 0.05). We observed the similar tendency of HIF-1*α* expression at protein level (Figure 4(c)).

VEGF expression in the DMH group or DMH + E_2_ group was higher than that in control group at both mRNA (Figure 4(b)) and protein (Figure 4(c)) levels. When we compared DMH + E_2_ group with DMH group, we found that estrogen treatment caused a significant decrease (1.43-folds) in VEGF mRNA expression (Figure 4(b); *P* < 0.05). The VEGF protein levels changed in the similar tendency (Figure 4(c)).

## 4. Discussion

Epidemical studies showed that postmenopausal women were at increased risk of colorectal cancer (CRC) compared with premenopausal women, and data from prospective randomized trials showed that HRT reduced the risk of CRC in postmenopausal women by 30 to 40% [18]. Many studies indicated that estrogen could inhibit proliferation and induce apoptosis in colon cancer cells [19, 20]. However, the study of estrogen on tumorigenesis of colon cancer in animal models was scarce. In the present study, we investigated the effect of estrogen on colon polyp formation in an ovariectomized rat model. As a result, we found that the multiplicity and volumes of polyps in the estrogen-DMH simultaneous treated group were lower than those in the DMH treated group. This result confirmed that estrogen could inhibit colon carcinogenesis in DMH-induced rat colon cancer model. And this result was accordant with the epidemiological results and the results found in colon cell models.

In our study, we found that the PCNA index of polyps in DMH + E_2_ group was significantly lower than that in DMH group. This result suggested estrogen could inhibit tumor formation by inhibiting the proliferation ability of colon stimulated by the carcinogen. This result was also in agreement with the findings in the cell model [19] which indicated estrogen could inhibit the proliferation of colon cancer cell. Many studies had demonstrated that estrogen could negatively regulate cellular proliferation by estrogen receptor beta in several types of cancers, such as ovarian cancer and prostate cancer [21, 22]. As estrogen receptor beta was the dominant estrogen receptor subtype in the colon tissue of rats [5], it was possible that estrogen might exert its antiproliferative effect by estrogen receptor beta in rats.

For the growth of tumor, increased proliferation must be accompanied by increased blood supply and this is achieved by angiogenesis and increased blood microvessel density [23]. Tumor angiogenesis is regulated by various activating and suppressive factors. Among the activating factors, VEGF plays a central role in the induction of angiogenesis. As an important transcription factor, HIF-1*α* could mediate the induction of VEGF expression. Estrogen played important roles in angiogenesis in healthy or pathological conditions. During menstrual cycle, estrogen stimulates angiogenesis in the uterine endometrium. While in breast cancer, estrogen was found to induce the expression of VEGF [24]. However, the effects of estrogen on angiogenesis or proangiogenic factors were distinct in different conditions. And the difference might be related to the different predominant subtype of estrogen receptors expressed in the tissues. For example, estrogen could induce the expression of VEGF and activation of HIF-1*α* in uterus mainly expressing estrogen receptor *α* [25], but estrogen inhibited angiogenesis and reduced the expression of VEGF in breast cancer which mainly expressed estrogen receptor beta [23]. In another study in prostate cancer [26], ER beta was reported to repress the transcription of VEGF and destabilize HIF-1*α*. These studies suggested that estrogen could inhibit angiogenesis and depress proangiogenic factors by ER beta. In our study, we found estrogen significantly depressed the microvessel densities in DMH-induced colon polyps. Accordingly, our study indicated that estrogen reduced the expression of VEGF and HIF-1*α* in DMH-induced colon polyps. As ER beta was also the predominant ER subtype in the colon of rats, we hypothesized that estrogen might inhibit angiogenesis of colon polyps by downregulating HIF-1*α* and VEGF via ER beta.

In summary, the present study demonstrated that estrogen could inhibit colon polyp formation in a rat model of colon cancer induced by DMH. Meanwhile, estrogen depressed the microvessel densities and reduced the expression of VEGF and HIF-1*α*. We supposed that estrogen might inhibit colon carcinogenesis by downregulating HIF-1*α* and VEGF, eventually reducing angiogenesis.

## Figures and Tables

**Table 1:** Primer sequences of HIF-1*α*, VEGF-A, and GAPDH genes.

Gene	Forward sequence	Reverse sequence
HIF-1*α*	CCTACTATGTCGCTTTCTTGG	GTTTCTGCTGCCTTGTATGGG
VEGF-A	CAGCTATTGCCGTCCAATGA	CCAGGGCTTCATCATTGCA
GAPDH	ACAGCAACAGGGTGGTGGAC	TTTGAGGGTGCAGCGAACTT

**Table 2:** The incidence, multiplicity, average volume, and distribution of colon polyps.

Group	Amount of rats	Incidence of polyps	Polyp multiplicity	Average volume	Distribution of polyps	
					Distal part	Proximal part
Control	12	0	—	—	—	—
DMH	12	91.7% (11/12)	6.8 ± 3.1	102.97 ± 77.67	82.9% (68/82)	17.1% (14/82)
DMH + E_2_	12	66.7% (8/12)	3.0 ± 1.1*	45.85 ± 43.40*	72.2% (26/36)	27.8% (10/36)

*P < 0.05 compared with the DMH group.
